# Fusion of Raman and FTIR Spectroscopy Data Uncovers Physiological Changes Associated with Lung Cancer

**DOI:** 10.3390/ijms252010936

**Published:** 2024-10-11

**Authors:** Harun Hano, Beatriz Suarez, Charles H. Lawrie, Andreas Seifert

**Affiliations:** 1 CIC nanoGUNE BRTA, 20018 San Sebastián, Spain; 2 Department of Physics, University of the Basque Country (UPV/EHU), 20018 San Sebastián, Spain; 3 Faculty of Nursing and Medicine, University of the Basque Country (UPV/EHU), 48940 Leioa, Spain; beatriz.suarez@ehu.eus; 4 Biogipuzkoa Health Research Institute, 20014 San Sebastián, Spain; charles.lawrie@bio-gipuzkoa.eus; 5 IKERBASQUE—Basque Foundation for Science, 48009 Bilbao, Spain; 6 Sino-Swiss Institute of Advanced Technology (SSIAT), University of Shanghai, Shanghai 201800, China; 7 Radcliffe Department of Medicine, University of Oxford, Oxford OX3 9DU, UK

**Keywords:** data fusion, vibrational spectroscopy, chemometrics, feature selection, photonic diagnostics

## Abstract

Due to the high mortality rate, more effective non-invasive diagnostic methods are still needed for lung cancer, the most common cause of cancer-related death worldwide. In this study, the integration of Raman and Fourier-transform infrared spectroscopy with advanced data-fusion techniques is investigated to improve the detection of lung cancer from human blood plasma samples. A high statistical significance was found for important protein-related oscillations, which are crucial for differentiating between lung cancer patients and healthy controls. The use of low-level data fusion and feature selection significantly improved model accuracy and emphasizes the importance of structural protein changes in cancer detection. Although other biomolecules such as carbohydrates and nucleic acids also contributed, proteins proved to be the decisive markers found using this technique. This research highlights the power of these combined spectroscopic methods to develop a non-invasive diagnostic tool for discriminating lung cancer from healthy state, with the potential to extend such studies to a variety of other diseases.

## 1. Introduction

Lung cancer remains the most common cause of cancer death worldwide, with around 1.8 million deaths per year [1]. The prognosis is particularly poor, with a relative 5-year survival rate, as the diagnosis is made at a late stage, which limits the targeted treatment options [2]. Conventional diagnostic methods such as computed tomography (CT), sputum cytology, biopsy and bronchoscopy are often inadequate for early detection due to their cost, time-consuming nature and lack of sensitivity. Such limitations have paved the way for modern computer-aided diagnostic techniques, which represent a promising alternative as they offer less invasive and more patient-friendly methods without compromising diagnostic accuracy. These techniques have several advantages, including error reduction, faster results, greater efficiency and high reproducibility [3,4].

In recent years, the ability to collect large datasets with modern analytical instruments has proven extremely useful for analyzing, measuring and monitoring various biosamples [5]. In this context, vibrational spectroscopy, particularly Raman and Fourier-transform infrared spectroscopy (FTIR), stands out because of its high sensitivity to changes at the molecular level. These techniques are non-invasive, non-destructive and free of reagents and waste and provide detailed information on the composition and structural conformation of certain types of molecules [6]. FTIR spectroscopy measures the absorbance of infrared light by a sample, revealing insights into molecular vibrations, chemical bonds and functional groups. In contrast, Raman spectroscopy measures the inelastic scattering of light and provides complementary information of the molecular structure of the biosample [7].

It has been shown that the integration of data from Raman and FTIR spectroscopy using data-fusion techniques can significantly improve the robustness and accuracy of prediction models [8]. Raman spectroscopy detects subtle molecular vibrations of molecules with polarizability, while FTIR spectroscopy provides detailed information about molecular bonds and functional groups that have a permanent dipole moment. By merging these datasets, researchers can achieve a unified view of the molecular composition, leading to more accurate and reliable analytical outcomes [9]. The combination of Raman and FTIR measurements can even be accomplished in a single spectroscopic instrument that combines both techniques such that the sample can be measured at the same time and same position by both spectroscopic methods [10]. Data-fusion strategies can be categorized into three types: low-level, mid-level and high-level data fusion. In low-level data fusion (LLDF), data matrices from several sources are directly linked together to create a comprehensive dataset that covers the entire range of measured variables. Mid-level data fusion (MLDF) addresses the problem of high dimensionality by selecting or reducing features from the data prior to fusion, reducing data complexity while preserving important information and enabling more efficient model training. High-level data fusion (HLDF) combines the predictive results of models developed for each data source and improves predictive accuracy by leveraging the strengths of each individual model [9,11,12].

Here, we present new developments for an effective computer-aided diagnostic approach for lung cancer by integrating Raman and FTIR spectroscopy with advanced data-fusion techniques. This research aims to identify the most indicative and discriminative biomolecular groups for the detection of lung cancer. The data from both spectroscopy techniques are combined to utilize the complementary information they provide to develop more reliable prediction models. Data fusion is applied and assessed for all three levels, low-, mid- and high-level fusion techniques. The results are further improved by implementing specific feature-selection methods.

## 2. Results and Discussion

### 2.1. Model Performance

Table 1 presents the performance of various configurations using Raman and FTIR spectroscopy data, and highlights the impact of FS, FR and different data-fusion strategies on model performance. A critical evaluation is carried out here, focusing on the most important comparative findings.

**Figure 1:**
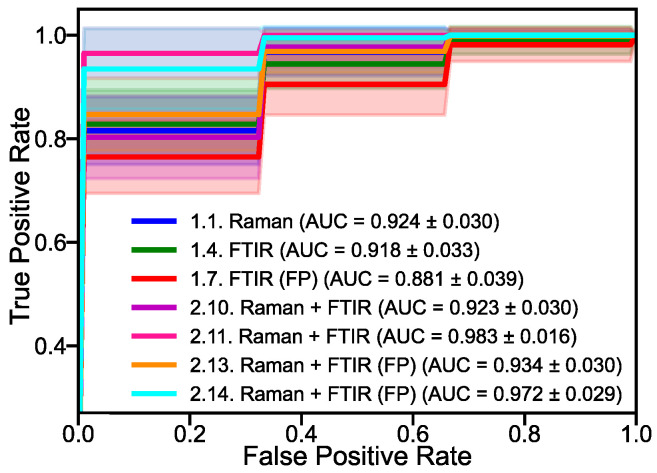
AUC-ROC scores for the selected data-fusion configurations, corresponding to Table 1.

Without data fusion, Option 1, Raman spectroscopy consistently outperforms FTIR in terms of accuracy. Specifically, Raman spectroscopy with FS achieves a notably higher accuracy of 0.85 with fewer features compared to the full spectral range (0.81) and feature reduction (FR) (0.84). Similarly, FTIR spectroscopy saw its base accuracy improve from 0.79 to 0.84 with FS, while FR yields a comparable result (0.79). These results highlight the superior performance of FS in isolating the most relevant features and reducing noise, thus significantly enhancing model accuracy. Conversely, FR simplifies data complexity and provides moderate improvements but may overlook some vital information. The area under the curve (AUC) of the receiver operating characteristics (ROC), shown in Figure 1, further supports these accuracy results. Raman spectroscopy achieves the highest AUC value of 0.92, exceeding the AUC value for the entire spectral range of FTIR at 0.92. Remarkably, the fingerprint region (FP) in FTIR achieves an AUC of 0.88. Although these values are somewhat lower, the proximity of these values indicates that a significant portion of the diagnostic information in FTIR likely comes from the fingerprint region, indicating that while the full spectral region provides a broader context, the fingerprint region alone captures most of the critical diagnostic features. It is important to note that for Raman spectroscopy, the spectra were only collected in the spectral range of 610–1720 cm^−1^, which is considered to contain most of the biological information and effectively serves as the fingerprint region in this context, whereas in FTIR, we specifically distinguished between the full range (Full: 400–4000 cm^−1^) and the fingerprint region (FP: 400–1800 cm^−1^) as indicated in Table 1.

LLDF, Option 2, which combines spectral Raman and FTIR data, demonstrates significant enhancements in model accuracy. When using the combined full spectral range, the accuracy reaches 0.86. Applying FS remarkably improves the accuracy to 0.99 with a substantially reduced number of features, highlighting the significant impact of FS in extracting the most discriminative information from both datasets. In contrast, FR achieves an accuracy of 0.87, which is higher than using individual methods but not as high as FS. These results indicate that FS is more effective than FR in LLDF, as it accurately isolates critical features and thereby significantly improves model performance. The AUC-ROC values in Figure 1 also show an improvement in accuracy. For the fused data, AUC calculates to 0.92, which increases substantially to 0.98 when applying FS. Moreover, the fingerprint region (FP) alone demonstrates notable AUC values of 0.93 and 0.97 using FS. These high AUC values show that the combination of spectral Raman and FTIR data, especially in feature selection, significantly improves the discriminatory power of the model. The strong performance of the fingerprint region suggests that it contains most of the critical diagnostic features, making it a valuable component for improving prediction accuracy.

MLDF, Option 3, where FS or FR is applied to both Raman and FTIR datasets before they are combined, shows remarkable improvements in model performance. FS achieves an accuracy of 0.85, which illustrates a significant increase in performance through the selection of critical features. Interestingly, in the FP region, FR proves more effective than FS, achieving an accuracy of 0.86 compared to 0.80. Evidently, FR captures better essential information with fewer components in specific spectral regions. These results emphasize the importance of tailoring dimensionality-reduction techniques to the characteristics of the data, as FR appears to be better able to distill critical information into a reduced feature set in the FP region. MLDF thus benefits from a differentiated application of dimensionality-reduction strategies, whereby the choice between FS and FR should be determined by the specific data characteristics.

HLDF, Option 4, combines the predictions from individual models trained on the separate Raman and FTIR data blocks by averaging their predicted probabilities. For HLDF, the combination of the entire spectral range achieves an accuracy of 0.84. FS gives a slightly lower accuracy of 0.81 with fewer features, while FR maintains the same level of accuracy. In the FP region, both FS and FR achieve a similar accuracy of 0.83, indicating that the choice between FS and FR depends on the dataset and spectral range. These outcomes show that HLDF is an effective strategy for integrating predictions from different data sources, although the optimal dimensionality-reduction technique must be tailored to the specific characteristics of the data. Neither FS nor FR were consistently better than the others, underlining the importance of dataset-specific assessment.

### 2.2. Graphical Representations

**Figure 2:**
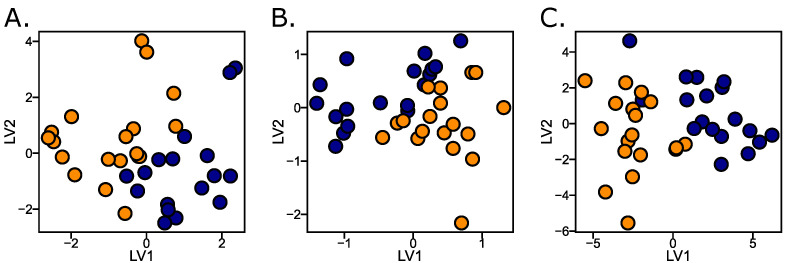
Score plots of (**A**) Raman, (**B**) FTIR and (**C**) the combined data from Raman and FTIR. Color codes are: orange for lung cancer patients and dark blue for healthy controls.

The high performance of the model observed in Section 2.1 is visually explained by the score plots in Figure 2. Both Raman and FTIR show a clear class separation, each with a unique pattern, indicating a strong discriminating power. The combined score plot after data fusion and block scaling shows an even clearer separation of classes. The score plots do not reflect the whole truth, as only a pattern with greatly reduced information can be visually represented, but it can be seen that data fusion improves the classification for linear models and thus the predictive power. By integrating the complementary data with high holistic information from Raman and FTIR spectroscopy, this approach provides detailed and reliable spectroscopic information, making it a valuable tool for cancer diagnosis.

### 2.3. Biochemical Assignment

**Figure 3:**
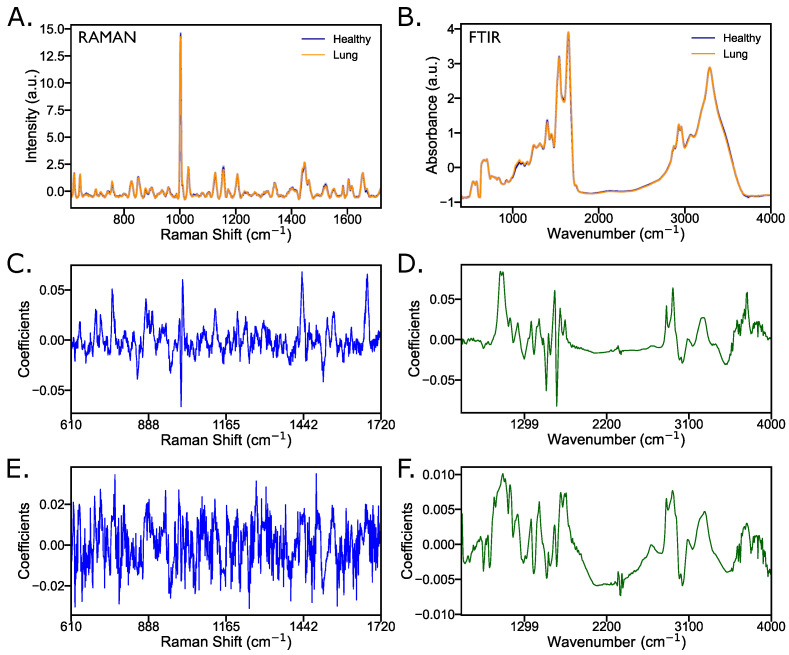
Raman and FTIR spectra with mean and standard error (**A**,**B**) and corresponding regression coefficients before (**C**,**D**) and after (**E**,**F**) block scaling, illustrating the impact of this technique on spectral data analysis. Color codes: Raman data in blue (**C**,**E**), FTIR data in green (**D**,**F**).

Figure 3 illustrates the vibrations assigned to specific biomolecular groups, showing their contribution to the observed separation and highlighting their significance in cancer diagnosis. The regression coefficients in Figure 3C–F provide insights into the specific molecular features responsible for the class separation and allow a deeper understanding of the underlying biochemical differences captured by each spectroscopic technique. Standard error in Figure 3A,B makes the variation in the spectra appear very small, almost invisible, indicating that the variability in the mean values is much smaller. This occurs because the standard error measures the precision of the sample mean—how much the sample mean is expected to vary from the true population mean—rather than the spread of individual data points.

**Figure 4:**
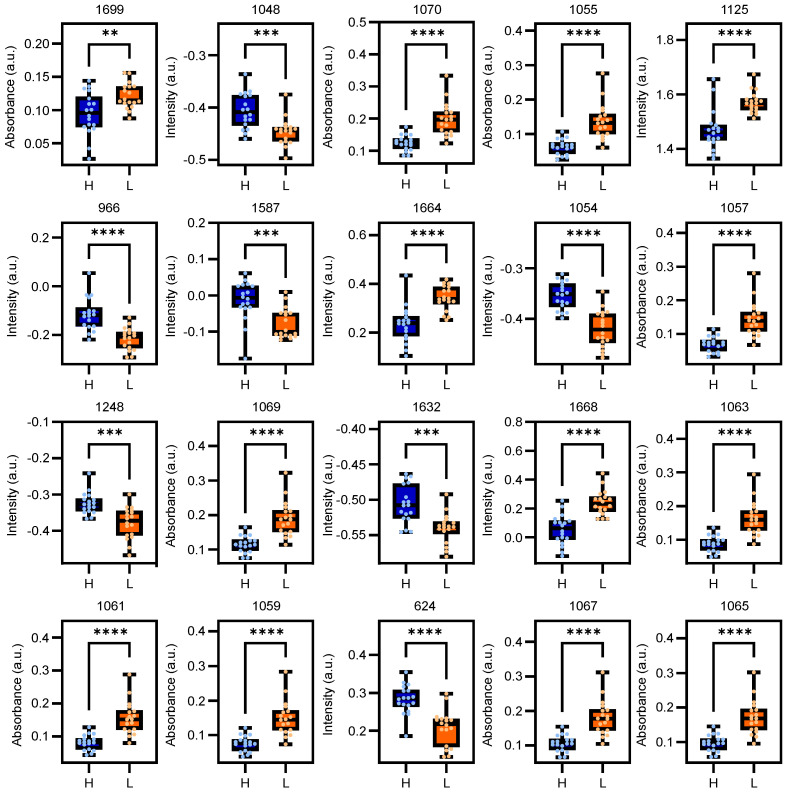
Box−and−whisker plots representing the top 20 wavenumbers obtained through data fusion, arranged in descending order of feature importance. All features show statistically significant differences between healthy controls (H) and lung cancer patients (L). Statistical analysis was performed using the Mann–Whitney U test in GraphPad Prism version 10.2.1 for Windows. ** *p* ≤ 0.01, *** *p* ≤ 0.001, **** *p* ≤ 0.0001.

High model performance observed in Section 2.1 can be attributed to the most important features identified by these two spectroscopic methods. As shown in Figure 3, the regression vectors provide an overall scheme for these differences and highlight the key biochemical groups that contribute to segregation. The analysis of these vectors shows which specific molecular characteristics are most influential for the differentiation between the classes. Box-and-whisker plots based on statistical analysis per wavenumber, as shown in Figure 4 and as assigned in Table S1 in the Supplementary Information [13,14,15,16,17,18,19], show the most significant features (wavenumbers) selected for their high predictive power by LLDF of Raman and FTIR with FS, as found by method 2.11 in Table 1. From the initial 173 features selected, recursive feature elimination (RFE) was applied to narrow down to the most important 20 features. These plots show the distribution of specific biochemical groups, providing insight into their role in class separation. The reason for focusing on these 20 features is that this selection method yields the highest performance, as demonstrated by the results in Section 2.1.

For Raman spectroscopy, peaks at 624 cm^−1^ are associated with C–C twisting in lipids and proteins (phenylalanine). The peak at 966 cm^−1^, linked to CH_3_ deformation, ring breathing, C–C stretching in proteins (tryptophan, valyl, prolyl) and lipids, is among the most critical features. Peaks between 1048–1054 cm^−1^ associated with =CH bending, C–C and C–O stretching in proteins (phenylalanine), collagen and glycogen, respectively, were highlighted as the most significant. The peaks at 1125 cm^−1^ also stand out due to C–C, C–O, C–N stretching in lipids, glycogen and proteins in lung cancer detection. The peak at 1248 cm^−1^ (protein C–N stretching) is significant for understanding biochemical differences. The peak at 1587 cm^−1^, corresponding to C=C stretching in tryptophan, highlights protein structural changes. In addition, the amide I bands from proteins, found at 1632–1668 cm^−1^, indicate changes in protein secondary structure, such as alpha-helices and beta-sheets, which are crucial for distinguishing lung cancer.

For FTIR spectroscopy, the vibrational band between 1055–1070 cm^−1^ is particularly significant. This range includes phosphate stretching bands, such as symmetric vibrations of ${\mathrm{PO}}_{2}^{-1}$ in phospholipids, and C–O symmetric vibrations. Moreover, the strong absorption band at 1699 cm^−1^ corresponds to C=O stretching vibrations in amide I, indicating significant protein changes in cancer patients. These specific vibrations originate from the fingerprint region, although the entire spectral range was used, which emphasizes the importance of this region for cancer diagnostics.

While various biochemical groups may be significant for distinguishing between healthy controls and lung cancer patients, protein-related vibrations are particularly important. Peaks at 1125 cm^−1^, 1587 cm^−1^ and 1632–1668 cm^−1^ in Raman spectroscopy as well as the strong absorption at 1055–1070 cm^−1^ and 1699 cm^−1^ in FTIR spectroscopy underline the crucial importance of protein structure changes in cancer diagnostics. Although other biomolecules such as carbohydrates and nucleic acids are also important, proteins appear to be the most important biochemical markers for accurate cancer detection, highlighting their crucial role in the diagnostic process.

## 3. Materials and Methods

### 3.1. Sample Collection and Preparation

In this study, 36 human subjects were examined: 18 healthy controls and 18 patients diagnosed with non-small cell lung carcinoma (NSCLC). Blood samples from NSCLC patients were collected at the Oncology Department of Donostia University Hospital (San Sebastián, Spain) and plasma was separated within one hour according to standard protocols. Additionally, plasma samples from 18 healthy donors were obtained retrospectively from the Basque Biobank, San Sebastián, Spain.

Raman analysis was performed by applying only 1 µL of human blood plasma from each subject to a slide-mounted aluminum foil and air-dried for 5 min. Aluminum foil was chosen for its high reflectivity, stability, flexibility, low background signal and cost efficiency, making it an ideal substrate for Raman signal amplification [13]. In contrast, the FTIR analysis was performed with an attenuated total reflectance crystal (ATR) as a contact sample method. A 1 µL aliquot of human blood plasma, corresponding to the amount used in the Raman analysis, was placed on the ATR crystal. The samples were completely dried before data collection to ensure consistent and reliable measurements.

### 3.2. Data Collection and Preprocessing

Raman measurements were performed with the Renishaw confocal Raman microscope inVia^TM^, Wotton-under-Edge, England, UK, operated at a laser wavelength of 785 nm, laser power of 73 mW, a 50× long-distance objective for focusing the laser beam and collecting Raman signals and a spectrometer grating of 1200 L/mm. This setup was selected to optimize Raman signals and signal-to-noise (SNR) ratio while minimizing sample damage. Each sample underwent 20 accumulations, each lasting 1 s. Spectra were acquired from 25 different points at the droplet’s periphery to capture the information from the high concentration of biomolecules that appear at the outer ring due to the coffee ring effect. Random cosmic ray interference was eliminated using the zap function in the Renishaw WiRE 5.4 software. Two preprocessing techniques were applied: asymmetric Whittaker baseline correction (λ=100, p=0.01) to remove baseline drifts and distortions, and standard normal variate (SNV) transformation to correct for variations due to sample thickness, scattering and instrumental response. Finally, 25 spectra per subject were averaged to obtain a single representative spectrum per subject. The averaging reduces random noise and improves SNR, providing a clear and comprehensive spectral signature for each subject [13].

FTIR measurements were carried out using the Bruker Vertex 70 spectrometer, Billerica, MA, USA, in ATR mode. The spectral resolution was set to 4 cm^−1^ and the sampling time for each measurement was 100 s. To avoid interference from water bands, the spectra were recorded after the sample was completely dried to ensure consistent and reliable data. For each sample, 10 spectra were collected to guarantee the stability and reproducibility of the results. Only the SNV transformation was applied to the data, as a baseline correction was not necessary and had no physical reason. Finally, 10 spectra were averaged for each subject to obtain a single representative spectrum.

### 3.3. Data Fusion and Model Building

**Scheme 1:**
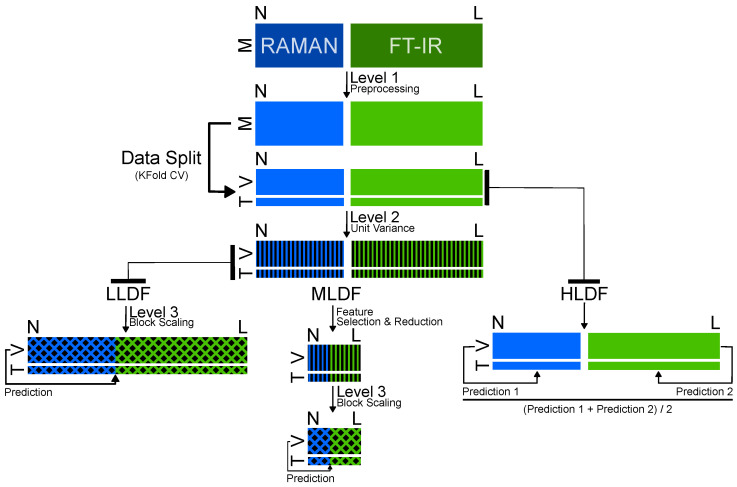
Schematic representation of multi-level data fusion of Raman and FTIR data for lung cancer detection.

The overall workflow shown in Scheme 1 describes the multi-level data-fusion strategy implemented in this study. It begins with row-based data preprocessing for Raman and FTIR spectra [20], as detailed in Section 3.2. The data are then partitioned into training and test datasets using stratified k-fold cross-validation with 6 folds, which was chosen as a trade-off between bias and variance due to the accuracy of the model (see Figure S1 in the Supplementary Information) and which mitigates overfitting by repeatedly partitioning the dataset into *k* subsets.

Later, unit variance scaling was applied to the preprocessed data after splitting. This column-based normalization technique adjusts the data so that each block has a unit variance and each variable within a block has the same variance equal to $1/n_{\mathrm{block}}$, where $n_{\mathrm{block}}$ is the number of variables in the respective block. This method is particularly useful for datasets that contain variables with different units or scales, as it ensures that all variables contribute equally to the analysis [20,21,22].

In LLDF, the data matrices of the individual methods were directly concatenated after the preprocessing steps mentioned above in order to optimize the variations within the block and to cover the entire range of measured variables. Soft block scaling was applied by adjusting each data column with a scaling factor to ensure uniformity across a combined dataset. This factor was calculated for each column as the standard deviation of the column multiplied by the fourth root of the number of features. With this method, each scaled column has the same variance, and the sum of their variances is equal to the square root of the number of variables in the block. This approach balances the influence of individual blocks, particularly, different dynamics in the data, prevents larger blocks from dominating the analysis and ensures an equal contribution from all blocks [20,23,24].

In contrast, MLDF requires feature selection (FS) or feature reduction (FR) before block scaling. This approach retains the most important features of each method, reducing data complexity while preserving pertinent information. In MLDF, the identified key characteristics were fused and block-scaled as described above to ensure the consistency and balance of the contributions of all variables. The following steps outline the process: 1. FS was performed using regression coefficients (RCs) from partial least squares regression (PLSR). Initially, features were ranked based on the absolute values of their RCs, arranged from highest to lowest. This systematic approach involved 100 iterations with 6 folds and makes sure that each feature was assessed 600 times. Features were selected in each iteration, based on specific percentiles, such as 1%, 2%, 3% and up to 10%. The selection frequency for each feature was calculated by counting how often it was selected across all iterations for each percentile. This process allowed us to identify the most significant features by their selection frequency. The features with the highest frequencies were then re-evaluated using the same percentile thresholds to ensure robustness. This method guarantees that only the most important features are retained. 2. FR was utilized with principal component analysis (PCA). The components were incrementally added, from 1 to 10, and model performance was calculated at each step. The highest accuracy was recorded with the specific combination of components that provided the best performance. This step ensures that the most informative components are used for model training, which further enhances the efficiency and effectiveness of the MLDF approach.

The most straightforward approach, HLDF, implies fusion of decisions and averages the predictions from individual data blocks, treating each block as equally important. Block scaling is therefore not an issue with HLDF, as no fused model per se, but only a fused decision is achieved; i.e., only the predictions of the individual models are combined [11]. Separate logistic regression models were trained for each method, and the predicted probabilities were then averaged. The final prediction was determined by applying a threshold to these averaged probabilities. Model performance was evaluated using accuracy scores, which were averaged across multiple folds for robust validation.

## 4. Conclusions

This study demonstrates the potential of combining Raman and FTIR spectroscopy with advanced data-fusion techniques to discriminate between healthy controls and lung cancer patients. By analyzing human blood plasma samples, we identified key protein-related vibrations that distinguish lung cancer patients, such as 1125 cm^−1^, 1587 cm^−1^ and 1632–1668 cm^−1^ in Raman spectroscopy and 1055–1070 cm^−1^, 1699 cm^−1^ in FTIR spectroscopy. These critical vibrational groups, identified through low-level data fusion supported by feature selection, significantly enhance model accuracy and highlight the importance of protein structural changes in cancer detection. Moreover, we were able to associate these spectral features with important biomarkers, providing insights into the underlying biochemical changes.

While other biomolecules such as carbohydrates and nucleic acids contribute to the classification, proteins were found to be the most decisive markers. The presented research underlines the potential of vibrational spectroscopy for the development of non-invasive diagnostic tools for the early detection of lung cancer. In particular, our research shows the power of a holistic approach—contrary to the analysis of single biomarkers—that takes into account the entirety of metabolic information that is accessible by Raman and FTIR spectrosopy.

This work provides a basis for further research into the molecular mechanisms of cancer and the development of new photonic diagnostic technologies based on large datasets that capture as much as possible the uncertainties associated with the method and the diversity of patients.

## Figures and Tables

**Table 1:** Comparison of various data-fusion methods and their accuracy.

Option	Data Fusion	Spectral Range for FTIR	Method	Data Range	Number of Features	Accuracy
1	No Fusion	Full	1. Raman	100%	1015	0.8119 ± 0.0035
			2. Raman (FS)	5%	51	0.8539 ± 0.0056
			3. Raman (FR)	6PCs	6	0.8378 ± 0.0060
			4. FTIR	100%	1868	0.7886 ± 0.0037
			5. FTIR (FS)	4%	75	0.8425 ± 0.0058
			6. FTIR (FR)	8PCs	8	0.7928 ± 0.0068
		FP	7. FTIR	100%	727	0.7567 ± 0.0033
			8. FTIR (FS)	1%	8	0.8419 ± 0.0057
			9. FTIR (FR)	5PCs	5	0.7633 ± 0.0068
2	LLDF	Full	10. Raman + FTIR	100%	2883	0.8625 ± 0.0035
			11. Raman + FTIR + FS	6%	173	0.9922 ± 0.0015
			12. Raman + FTIR + FR	6PCs	6	0.8711 ± 0.0034
		FP	13. Raman + FTIR	100%	1742	0.8592 ± 0.0037
			14. Raman + FTIR + FS	10%	175	0.9497 ± 0.0039
			15. Raman + FTIR + FR	5PCs	5	0.8681 ± 0.0031
3	MLDF	Full	16. Raman (FS) + FTIR (FS)	5% + 4%	126	0.8472 ± 0.0039
			17. Raman (FR) + FTIR (FR)	6PCs + 8PCs	14	0.8425 ± 0.0034
		FP	18. Raman (FR) + FTIR (FR)	5% + 1%	59	0.7972 ± 0.0035
			19. Raman (FR) + FTIR (FR)	6PCs + 5PCs	11	0.8583 ± 0.0032
4	HLDF	Full	20. Raman + FTIR	100%	1015/1868	0.8383 ± 0.0024
			21. Raman (FS) + FTIR (FS)	5%/4%	51/75	0.8131 ± 0.0023
			22. Raman (FR) + FTIR (FR)	6PCs\8PCs	6/8	0.8383 ± 0.0024
		FP	23. Raman + FTIR	100%	1015/727	0.8319 ± 0.0024
			24. Raman (FS) + FTIR (FS)	5%\1%	51/8	0.7989 ± 0.0035
			25. Raman (FR) + FTIR (FR)	6PCs \5PCs	6/5	0.8319 ± 0.0024

## Data Availability

The data presented in this study are available on request from the corresponding author.

## Supplementary Materials

The following supporting information can be downloaded at: https://www.mdpi.com/article/10.3390/ijms252010936/s1.
