# Supplementary material for: Fusion of Raman and FTIR Spectroscopy Data Uncovers Physiological Changes Associated with Lung Cancer
Source: Int J Mol Sci. 2024 Oct 11;25(20):10936. doi: 10.3390/ijms252010936 (PMC11507214; doi:10.3390/ijms252010936)
Supplement: Supplementary file 1 [file ijms-25-10936-s001.zip › ijms-3221787-supplementary.pdf]

Supporting Information

Fusion of Raman and FTIR Spectroscopy Data Uncovers Physiological Changes Associated with Lung Cancer

Harun Hano [1,2,*], Beatriz Suarez [3,4], Charles H. Lawrie [4,5,6,7] and Andreas Seifert [1,5,*]

1 CIC nanoGUNE BRTA, 20018 San Sebastián, Spain

2 Department of Physics, University of the Basque Country (UPV/EHU), 20018 San Sebastián, Spain

3 Faculty of Nursing and Medicine, University of the Basque Country (UPV/EHU), 48940 Leioa, Spain; beatriz.suarez@ehu.eus

4 Biogipuzkoa Health Research Institute, 20014 San Sebastián, Spain; charles.lawrie@bio-gipuzkoa.eus

5 IKERBASQUE—Basque Foundation for Science, 48009 Bilbao, Spain

6 Sino-Swiss Institute of Advanced Technology (SSIAT), University of Shanghai, Shanghai 201800, China

7 Radcliffe Department of Medicine, University of Oxford, Oxford OX3 9DU, UK

* Correspondence: h.hano@nanogune.eu (H.H.); a.seifert@nanogune.eu (A.S.); Tel.: +34-943-574-045 (A.S.)

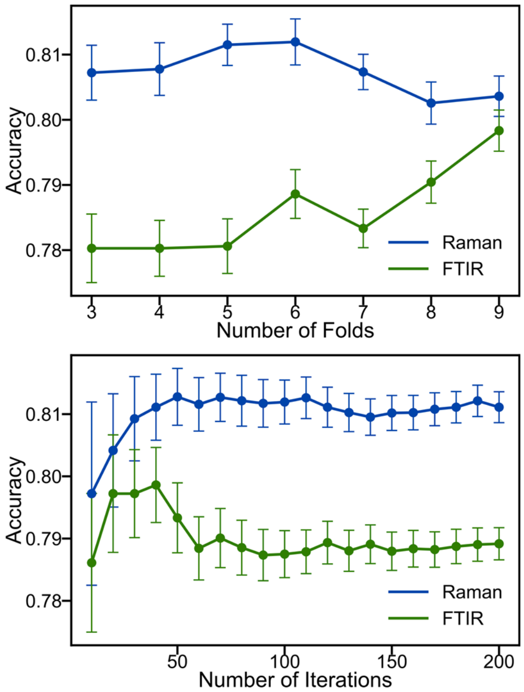

Figure S1. Choosing an optimized number of folds and iterations in terms of model accuracy for both Raman and FTIR data.

Table S1. Raman and FTIR spectral band assignments for human blood plasma [13–19].

**Raman**		**FTIR**	
Peak Positions	Vibrational Modes	Peak Positions	Vibrational Modes
619-624	τ(CC): Phe	579	C=O bending
641-643	τ(CC): Tyr	700	N–H bending
698-701	ν(CS): Met	833-1078	ν_s(C–O); $\nu_s(PO_2^-)$
756-758	δ(CH)ring: Trp	1169	ν_{as}(C–O): esters
822	δ(CH)ring: Tyr	1246	Amide III; $\nu_{as}(PO_2^-)$
855-856	δ(CH)ring: Tyr	1315	Amide III
874-878	ν(CN), ν(CC): proteins, lipids; Trp, Arg	1400-1450	$\delta(CH_3)$: proteins/side chains
897-901	ν(CC), ν(CN)	1537	Amide II
939	ν(CC), ν(CN), $\delta(CH_2)$	1651-1699	Amide I
966	CH_3 deformation, ring breathing, ν(CC)	1740	ν(C=O): lipids
1000-1004	ν_s(C–C)ring: Phe	2868	$\nu_s(CH_2)$: lipids
1029-1033	ρ(C–H): phenylalanine	2930	$\nu_s(CH_2)$: lipids
1048-1054	δ(=CH), ν(CC), ν(CO)	2961	$\nu_{as}(CH_3)$: lipids
1104	ν(C–C): the gauche-bonded chain	3071	Amide II, proteins
1123-1127	ν(C–C): phospholipids	3294	Amide I, ν_s(N–H), and OH groups
1156-1157	ν(CC): carotenoids		
1204-1210	δ(CH), τ(CH2)		
1232-1269	δ(NH), ν(CN): amide III in proteins		
1397-1404	$\nu_s(COO^-)$: Asp, Glu		
1436-1438	$\delta_{as}(CH_3)$, $\delta(CH_2)$		
1513-1528	ν(C=C): carotenoids		
1548-1553	Tryptophan		
1587-1589	ν(C=C)		
1604-1606	ν(C=C): phenylalanine and tryptophan		
1619	ν(C=C): tyrosine and tryptophan		
1666-1671	Amide I: $\alpha - helix$		
